# Effect of repeated hot water immersion on muscle strength, power, function and physical activity in healthy older adults: A randomised crossover trial

**DOI:** 10.1113/EP093501

**Published:** 2026-04-20

**Authors:** Daniel D. Piccolo, Jo Corbett, Timothy A. Exell, Joseph M. Moore, Amy Wright, Mohammad G. A. Alnajjar, Luke C. Hudson, Poppy A. Marsh, Veronika Praskacova, Melitta A. McNarry, Kelly A. Mackintosh, Zoe L. Saynor, Anthony I. Shepherd

**Affiliations:** ^1^ Clinical, Health and Rehabilitation Team, Centre for Integrated Health and Wellbeing, School of Psychology, Sport and Health Sciences, Faculty of Science and Health University of Portsmouth Portsmouth UK; ^2^ Extreme Environments and Occupational Performance Group, School of Psychology, Sport and Health Sciences, Faculty of Science and Health University of Portsmouth Portsmouth UK; ^3^ Applied Sports, Technology, Exercise and Medicine (A‐STEM) Research Centre, School of Sport and Exercise Sciences Swansea University Swansea UK; ^4^ School of Health Sciences Faculty of Environmental and Life Sciences University of Southampton Southampton UK; ^5^ National Institute for Health and Care Research Southampton Biomedical Research Centre Southampton UK

**Keywords:** ageing, grip strength, lower body kinetics, lower extremity function, passive heating

## Abstract

Ageing leads to an increased prevalence of sarcopenia and frailty, characterised by progressive declines in muscle strength, power and function and reduced physical activity. Hot water immersion (HWI) could potentially improve muscle function, but this is yet to be explored in older adults. Twelve middle‐aged to older adults completed a randomised, controlled, crossover trial (ClinicalTrials.gov ID NCT05618197), undergoing assessments before and after a 6‐week HWI intervention (two to three 60‐min HWIs per week) or control condition with a 6‐week washout between study arms. During HWIs, body position was adjusted to maintain rectal temperature at 38.5–39.0°C. Pre‐ and post‐intervention and control measurements of peripheral muscle strength (isokinetic and handgrip dynamometry), lower body power and functional performance (Short Physical Performance Battery consisting of balance, walking and sit to stand tests with motion and external force capture) and physical activity (accelerometry) were taken. Repeated HWI had no effect on the primary outcome peak quadriceps torque (*P *= 0.127, η^2^_p_ = 0.125; *n* = 7), whilst grip strength increased in the control arm (*P* = 0.004) and decreased post‐intervention compared to control (*P* = 0.039). SPPB total and component scores, lower body power, gait measures and physical activity levels were unchanged (all *P* > 0.05). Repeated HWI under the conditions employed did not improve strength, power, lower extremity function or physical activity levels in this cohort, and does not appear to be an effective method to improve indices of muscle function in healthy older adults.

## Abstract (highlights)

- **What is the central question of this study?**
  Can 6 weeks of two to three hot water immersions per week improve strength, power, lower extremity function and physical activity in healthy older adults?
- **What is the main finding and its importance?**
  Repeated hot water immersion had no effect on strength, power, lower extremity function or physical activity in healthy older adults. Six weeks of up to thrice‐weekly hot water immersion therefore does not appear to improve muscle function in healthy older adults, however, larger trials focusing on individuals with low physical function or frailty are warranted to confirm these findings.

## 1 INTRODUCTION

Ageing leads to an increased prevalence of sarcopenia (Cruz‐Jentoft et al., 2019) and frailty (Collard et al., 2012), which are characterised by progressive declines in muscle strength (Hughes et al., 2001), power (Runge et al., 2004) and functional capacity (Larsson et al., 2019). These changes can contribute to low levels of physical activity (Davis & Fox, 2007), increased risk of falls (Ambrose et al., 2013) and reduced walking speed (Dumurgier et al., 2009), which are associated with increased morbidity (Ambrose et al., 2013) and mortality (Dumurgier et al., 2009; Stanaway et al., 2011). Although regular exercise remains one of the most effective strategies to preserve and increase muscle size and improve function (Chambers et al., 2020), adherence among older adults can be poor due to physical limitations and contraindications (Stutts, 2002; Lees et al., 2005). With populations ageing rapidly worldwide (United Nations, 2024) and rising healthcare costs associated with muscle weakness (Pinedo‐Villanueva et al., 2019), there is an urgent need to identify accessible alternative interventions that can maintain or improve physical function later in life.

Passive heat therapy (PHT) has emerged as a promising adjunct treatment for cardiovascular (Oyama et al., 2013; Brunt et al., 2016) and metabolic health (Ely et al., 2019; James et al., 2023), with similar physiological mechanisms underpinning the benefits as traditional exercise (Cullen et al., 2020) which could improve muscle health (Rodrigues et al., 2020a). Despite its potential for beneficial muscle adaptations, few studies have focused on the effects of repeated PHT on muscle function in older adults. In younger and middle‐aged populations, repeated PHT interventions have previously been shown to improve muscle function. Repeated local thigh heating (diathermy) over 10 days attenuated immobilisation‐induced muscle atrophy (Hafen et al., 2019), while similarly, 8 (Kim et al., 2020) and 10 (Goto et al., 2011) weeks of thigh heating (water‐perfused trousers and steam‐generating sheet, respectively) increased strength and muscle cross‐sectional area. Repeated whole‐body passive heat acclimation (48–50°C climatic chamber) for 11 days increased strength and contractile function in young, healthy males, potentially via an increased rate of cross‐bridge formation (Racinais et al., 2017).

While repeated PHT appears to benefit muscle in younger and middle aged individuals, early experimental evidence in older adults is equivocal, showing that 8 weeks of sauna bathing (Fuchs et al., 2024) and 12 weeks of lower‐body PHT (water‐perfused trousers) (Ro et al., 2025) do not improve leg strength or muscle protein synthesis (Fuchs et al., 2024). Fuchs et al. (2024), however, observed a significant increase in grip strength, suggesting PHT's potential for a clinical benefit. A possible reason for a lack of improvement in leg strength could be that the modalities of repeated PHT previously examined (Fuchs et al., 2024; Ro et al., 2025), which did not prevent evaporative heat loss, did not provide a sufficient stimulus to improve muscle function in older adults, who have reduced muscle strength and mass. Hot water immersion (HWI) limits evaporative heat loss in the areas immersed and, in conjunction with the high thermal conductivity of water (Ramires et al., 1995), rapidly increases local tissue temperature (Rodrigues et al., 2020b) and blood flow (Amin et al., 2021). Therefore, whole‐body HWI could provide a greater stimulus and subsequent larger effect on multiple muscle groups and aspects of muscle function.

Accordingly, this study aimed to assess the effects of repeated (two to three times per week) HWI on indices of muscle strength, power, function and physical activity in older adults compared to a control period. We tested the null hypothesis that, in a cohort of healthy older adults, repeated HWI over 6 weeks would have no effect on (1) peak quadriceps strength, (2) grip strength, (3) knee extension power, (4) performance in a physical function testing battery, and (5) habitual physical activity.

## 2 METHODS

### 2.1 Ethical approval

This open label, randomised controlled, crossover trial was given a favourable ethical opinion by the University of Portsmouth Faculty of Science and Health Ethics Committee (SHFEC 2022‐054) and adhered to the *Declaration of Helsinki*. The study was pre‐registered on ClinicalTrials.gov (ID NCT05618197), with outcomes for the present study included as secondary outcome measures within a larger trial. Participants were supplied with a detailed study information sheet no less than 48 h before providing their written informed consent. A full verbal briefing was conducted, during which participants could ask questions about the study.

### 2.2 Participants

Participants were recruited through media releases, local interest groups, databases, posters and word of mouth. Inclusion criteria included: age ≥55 years, and free from cardiometabolic or musculoskeletal conditions that would contraindicate exercise. Exclusion criteria included: body mass index >35 kg m^−2^, uncontrolled hypertension (≥150 mmHg systolic blood pressure (BP) and/or ≥90 mmHg diastolic BP), current or recent smoking, and having spent prolonged time in a hot climate in the preceding 3 months. Participants also had refrained from using hot tubs or saunas for at least 6 weeks preceding the trial.

Participants attended an initial screening visit which included a health history questionnaire, and assessments of height, body mass, BP and a 12‐lead electrocardiogram. Eligible participants were randomised 1:1 (https://www.randomizer.org) to either start with the control (CON) or hot water immersion (HWI) condition. Arm 1 was followed by a 6‐week washout period, during which participants maintained their usual lifestyle, but refrained from new exercise programmes or heating interventions (e.g. saunas), and participants avoided hot baths for the full duration of the trial. Following the 6 weeks washout period, participants returned to complete the remaining condition.

The primary outcome was change in peak quadriceps torque, assessed via isokinetic dynamometry. The present study was part of a wider study which aimed for 13 older adults to complete the trial, and therefore we sought to recruit 20 people, to account for potential dropouts.

### 2.3 Study overview

Participants attended the laboratory and completed a baseline experimental visit 1–2 weeks before beginning their first allocated condition. The HWI arm of this trial consisted of 12–18 HWI sessions (two to three per week) in the Extreme Environments and Clinical, Health and Rehabilitation laboratory over 6 weeks, with post‐testing 48–72 h after the final session, in line with previous research in our lab (James et al., 2023). In the CON arm, participants continued their usual routines and returned to the laboratory for post‐testing 6‐week later.

### 2.4 Protocol

#### 2.4.1 Experimental visits

Participants arrived at the laboratory in the morning (10.00 h ±1 h), having abstained from heavy exercise and alcohol for 24 h and did not consume caffeine on the morning of the visit. After a measurement of maximal grip strength was taken, participants were instrumented with retroreflective markers, before undertaking assessments of quadriceps muscle strength, followed by a functional exercise test [Short Physical Performance Battery (SPPB); Guralnik et al., 1994].

#### 2.4.2 Intervention (hot water immersion)

During the intervention arm, participants were asked to complete two to three HWI sessions per week for 6 weeks, which is similar to previous studies implementing HWI interventions in healthy and clinical populations (Brunt et al., 2016; Hesketh et al., 2019; Oyama et al., 2013). During each HWI session, participants were immersed to just below shoulder height in 40°C water for 60 min in a hot tub (Hawaii, Lay‐Z‐Spa, Newton Abbot, UK), or temperature‐controlled immersion pool within the Extreme Environments Laboratory (University of Portsmouth). To continuously monitor deep body temperature (*T*_rec_) (2040 Squirrel, Grant Instruments, Cambridge, UK), participants self‐inserted a rectal thermistor (rectal temperature probe, Philips, Netherlands; YSI 400 series temperature probe, PROACT Medical, Corby, UK) to a depth of 15 cm. Body position was manipulated (i.e. the amount that was immersed) as necessary, to ensure participants reached and maintained a target *T*_rec_ of 38.5–39.0°C throughout each HWI. For safety purposes, heart rate was continuously monitored (FT1, Polar, Kempele, Finland), and BP was measured every 15 min (M3, Omron, Kyoto, Japan). Following each HWI session, participants laid on a semi‐recumbent bed and were free to depart the laboratory once *T*_rec_ had lowered to <38.5°C (13 ± 4 min post‐HWI).

### 2.5 Outcome measures

#### 2.5.1 Peak quadriceps torque

Quadriceps maximal isometric strength of the dominant leg was measured (verbally confirmed by each participant) using an isokinetic dynamometer (IKD) chair (HUMAC NORM, Computer Sports Medicine, Inc., Stoughton, MA, USA), according to the previously published protocol (Harbo et al., 2012). Specifically, participants were seated, with both knees and hips at 90° of flexion, their leg against the lever arm, and held in position with straps crossing the upper body and the hip. Participants performed two warm‐up familiarisation sets, where they were instructed to extend the knee into the lever arm. The first warm‐up set consisted of five submaximal contractions at a self‐selected 50% effort with 5 s rest between repetitions. Following 30 s rest, participants completed two submaximal contractions, at 75% effort with 20 s rest between repetitions. After a further 30 s rest, participants completed three maximum voluntary isometric contractions (MVIC), with each repetition separated by a 30 s rest. The highest torque achieved during any of the individual trials was recorded as the maximum.

#### 2.5.2 Handgrip strength

Handgrip strength, a global measure of strength and a powerful predictor of quality of life and mortality (Cruz‐Jentoft et al., 2019), was measured using a handgrip dynamometer (Takei 5001, Takei Scientific Instruments, Tokyo, Japan), following previously described methods (Roberts et al., 2011). Participants were seated in a chair, with their feet flat on the floor, and held the device by their side with their arm fully extended toward the floor. Three maximal trials on each side were performed, with the highest grip score from all six trials used. Rest between trials was self‐paced, with participants indicating when they were ready to perform the next trial.

#### 2.5.3 Short physical performance battery

To test for lower extremity function, the SPPB was administered, consisting of three components: a three‐part balance test, a 4‐m walk test and a chair sit to stand (STS) test. The SPPB is a low cost and reliable method for measuring lower extremity function in older adults (Guralnik et al., 1994) and a predictor of falls risk (Lauretani et al., 2019), hospitalisation (Studenski et al., 2003) and all‐cause mortality (Pavasini et al., 2016). Each individual component was scored out of 4, with the scores of the three tasks summed for a maximum total score of 12; a higher score is indicative of better physical performance. Participants first performed a balance test while standing on one force plate which consisted of three consecutive positions, first with feet together, second with feet semi‐tandem, and third with feet full tandem, with the aim to hold each position for 10 s. If participants were unable to complete the full 10 s of any stage of the balance test, the test was terminated, and they were moved on to the next component of testing. Participants subsequently performed two 4‐m walking trials at their normal walking pace with the time of the second trial scored. Participants then performed up to four further walking trials that were not scored in the SPPB for purposes of motion capture (see below). Finally, participants performed five maximal STS repetitions, beginning by sitting with their knees at 90°, arms crossed over their chest, and each foot on a force plate.

#### 2.5.4 Motion capture

**FIGURE 1:**
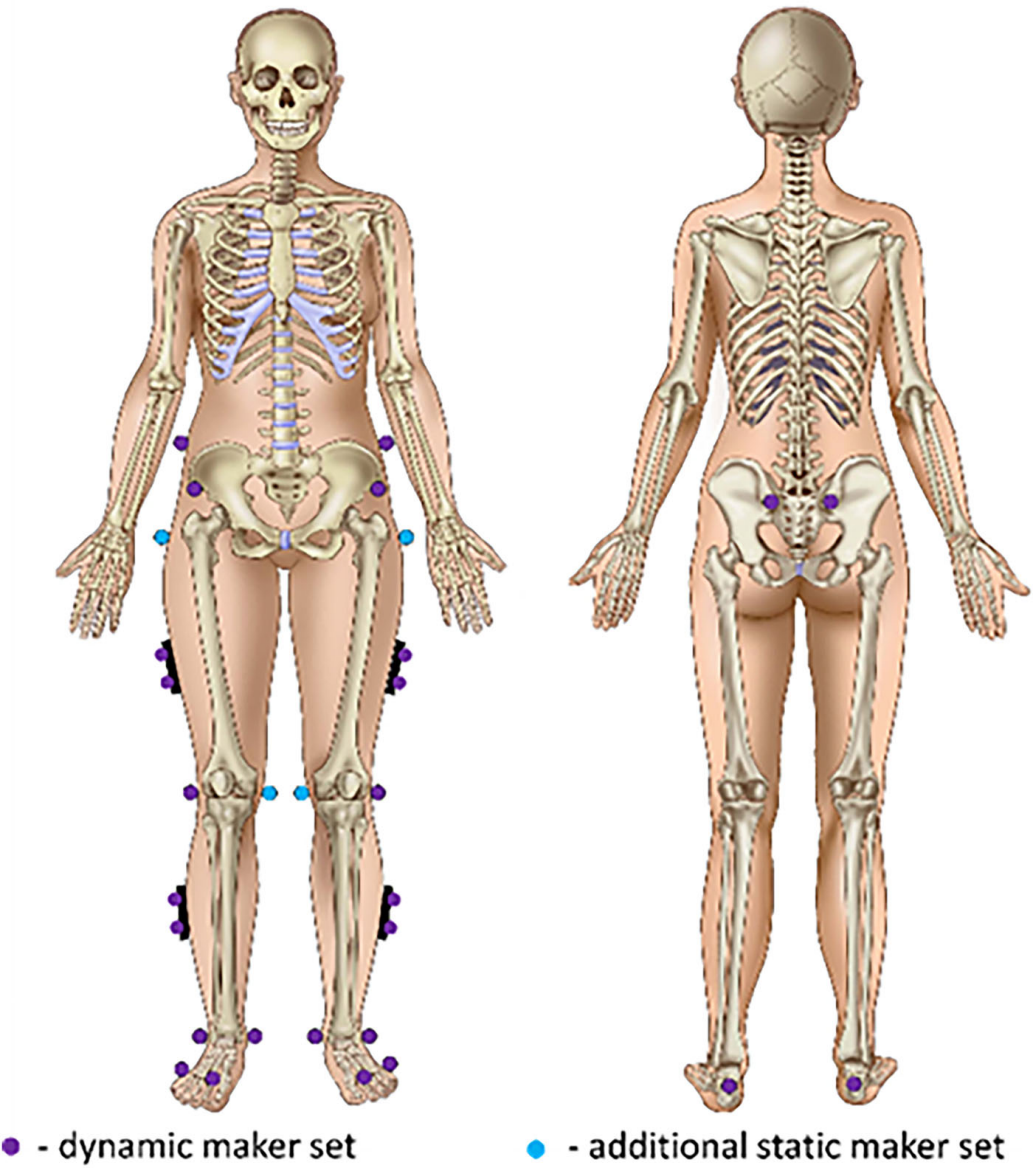
Marker placements for the measurement of lower limb kinematics and kinetics.

Motion capture and ground reaction force data were collected for analysis of lower body power and gait characteristics during the SPPB using a three‐dimensional optoelectronic system (200 Hz; 16 Oqus and Arqus cameras; Qualisys, Göteborg, Sweden), and two force plates (1000 Hz; Kistler 9281CA; Kistler, Winterthur, Switzerland), through Qualisys Track Manager software. All participants wore shorts and the same athletic trainers for all testing sessions. Retroreflective markers were placed on the skin and shoes, superficial to anatomical landmarks (Figure 1), to allow the measurement of pelvis, thigh, shank and foot position data. A static calibration trial was captured, with the participant standing in the anatomical position (Figure 1) before starting the SPPB.

After completing two walking trials for the SPPB, participants then completed one to four additional 4‐m walking trials to collect a successful foot strike on the force plate. For walking trials, marker position data were exported to MATLAB (2025a, The MathWorks, Natick, MA, USA). Gait was assessed for strides comprising two steps to remove the influence of step asymmetry. Stride length and frequency were defined as the antero‐posterior displacement of the right calcaneus marker between successive ground contacts and as the inverse of time between those ground contacts, respectively. Stride velocity was calculated as the product of stride length and frequency. Instants of ground contact were identified from the peak vertical acceleration of the calcaneus marker, whilst the marker was within 0.05 m of the minimum vertical position throughout the trial.

Data from STS trials were labelled and exported for analysis in Visual3D (HAS‐Motion, Kingston, Ontario, Canada) to calculate joint kinetics and provide insight into muscle function. Data were filtered using a zero‐lag low‐pass second‐order Butterworth filter with cut‐off frequencies of 6 and 15 Hz for marker and force data, respectively. A lower body model was created following Visual3D guidelines, and joint kinematics and kinetics were calculated. Peak knee extension velocity, moment and power were extracted for each concentric phase of the STS. The average across the three reps was used for further analysis.

#### 2.5.5 Habitual physical activity

Habitual physical activity (PA) was assessed using GENEActiv (Activinsights, Kimbolton, UK) accelerometers, which are accurate and reliable for assessing PA and sedentary time in adults (Esliger et al., 2011; Pavey et al., 2016). In the CON arm, participants wore the accelerometer during the first and final week. In the HWI arm, participants wore the accelerometer for a baseline 7‐day period before the first HWI session, and during the final week of HWI. The wrist worn GENEActiv accelerometers were set at a sampling frequency of 100 Hz and worn continuously over a period of 7 days. Following the measurement periods, data were downloaded using manufacturer's software and processed in R (version 4.2.2, R Core Team; R Foundation for Statistical Computing in Vienna, Austria), using the open source *GGIR* software package (http://cran.r‐project.org/package = *GGIR*). Previously validated acceleration threshold values were used to quantify the time (min day^−1^) spent on average in each PA intensity category (Fraysse et al., 2021). Participants who recorded <4 days with 16 h wear‐time were excluded.

### 2.6 Data analysis

Statistical analyses were conducted using SPSS Statistics, version 30.0 (IBM Corp., Armonk, NY, USA). Data are presented as means ± standard deviation (SD) unless otherwise stated, with statistical significance set at *P* < 0.05. Main and interaction effects for strength, lower body kinetics, gait measures and the SPPB were evaluated using a 2 × 2 linear mixed model, which accounted for missing data points within our lower body kinetics and accelerometry data (CON for *n* = 2, post‐CON for *n* = 2, post‐HWI for *n* = 1 for knee moment and knee power; CON for *n* = 2, post‐CON for *n* = 3 for all accelerometry variables). If residuals were not normally distributed, a generalised linear mixed model was performed. Significant interactions were followed by *post hoc* analysis with a Bonferroni correction. Mean differences and 95% confidence intervals were calculated for interaction and main effects. Effect sizes were reported as partial eta squared (η^2^_p_; small = 0.01, medium = 0.06 and large = 0.14) (Lakens, 2013). To assess potential order (i.e. learning) effects, a one‐way repeated measures linear mixed model (for parametric data) or generalised linear mixed model (for non‐parametric data) was conducted.

## 3 RESULTS

**FIGURE 2:**
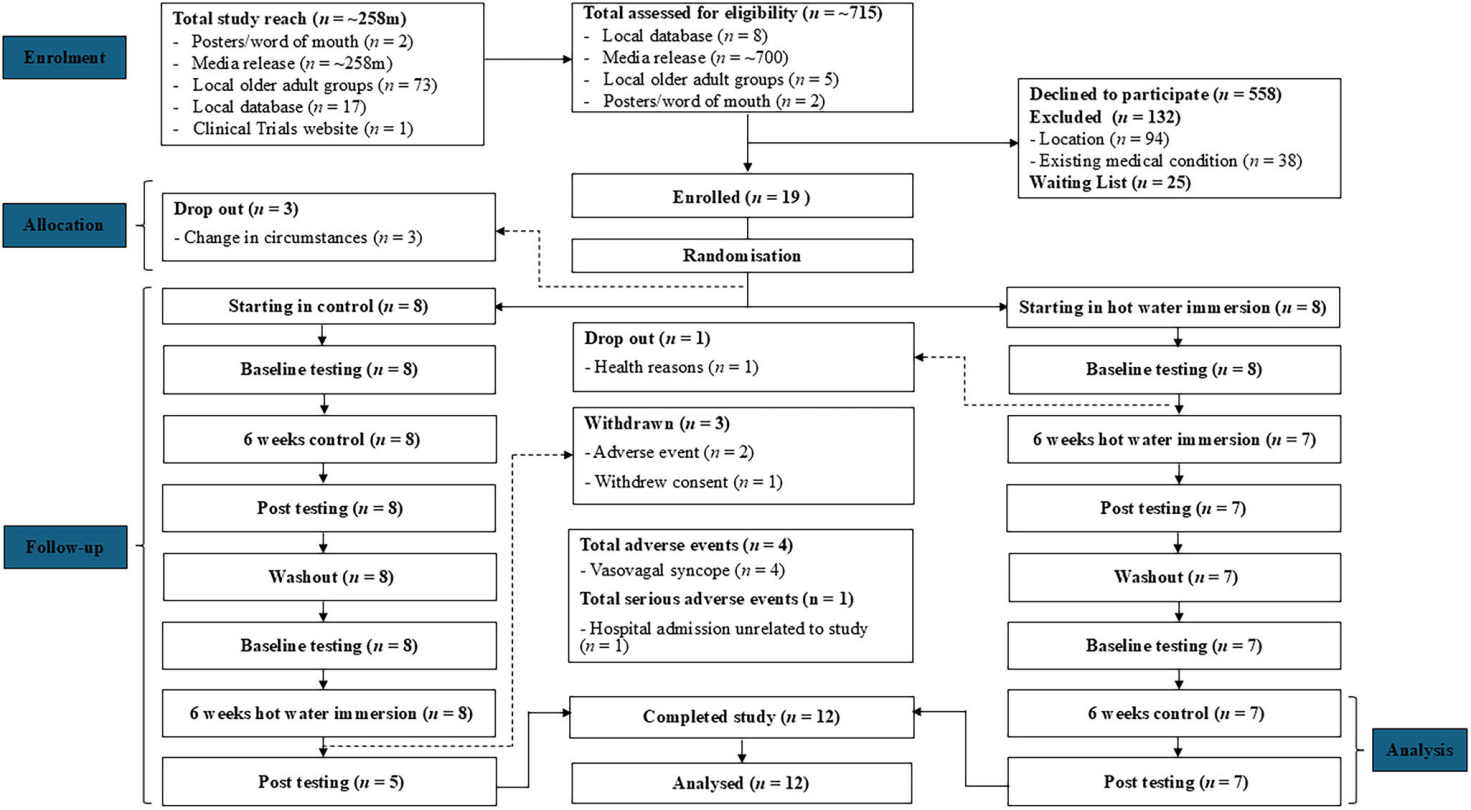
Participant flow through the trial.

Nineteen participants (Table 1) were recruited, of whom 12 completed the study (Figure 2). Attrition was due to change in personal circumstances (*n *= 3), health reasons (*n *= 1), withdrawn consent (*n *= 1), and two adverse events (*n *= 2), in which both participants withdrew following vasovagal syncope during venous cannulation that took place within the larger trial. Due to equipment failure during the trial, only seven participants completed the primary outcome measure of maximal quadriceps strength testing on the IKD, and therefore results for this outcome measure did not have adequate statistical power. Thermophysiological responses during the HWI intervention are detailed in full by (Piccolo et al., 2026). Grand mean starting and peak *T*_rec_ for each HWI session was 37.13 ± 0.19°C and 38.81 ± 0.09°C, respectively, and participants completed 13–18 HWIs, with all but one participant completing at least 15 sessions. The full anonymised dataset has been made freely available as supplementary material on our university repository (https://doi.org/10.17029/40adb0f3‐23ed‐48c0‐9cd3‐ff7083c9d2f4). Main and order effects tables are provided as an appendix (Tables A1, A2, A3, A4, A5, A6, A7, A8).

**TABLE 1:** Participant characteristics (*n* = 12).

Parameter	Value
Age (years)	69.2 ± 10.0
Males (*n*)	4
Females (*n*)	8
Height (cm)	168.9 ± 10.0
Mass (kg)	72.4 ± 16.8
BMI (kg m^−2^)	25.2 ± 4.1
SBP (mmHg)	131 ± 10
DBP (mmHg)	78 ± 7
Medications	
Alendronic acid (*n*)	1
Amitriptyline (*n*)	2
Amlodipine (*n*)	1
Beclometasone (*n*)	1
Finasteride (*n*)	1
Glucosamine (*n*)	1
Levothyroxine (*n*)	1
Salbutamol (*n*)	2
Simvastatin (*n*)	4
Solifenacin (*n*)	1
Tramadol (*n*)	1
Zopiclone (*n*)	1

*Note*: Data are expressed as mean ± SD unless otherwise stated.

Abbreviations: BMI, body mass index; DBP, diastolic blood pressure; SBP, systolic blood pressure.

### 3.1 Strength

**FIGURE 3:**
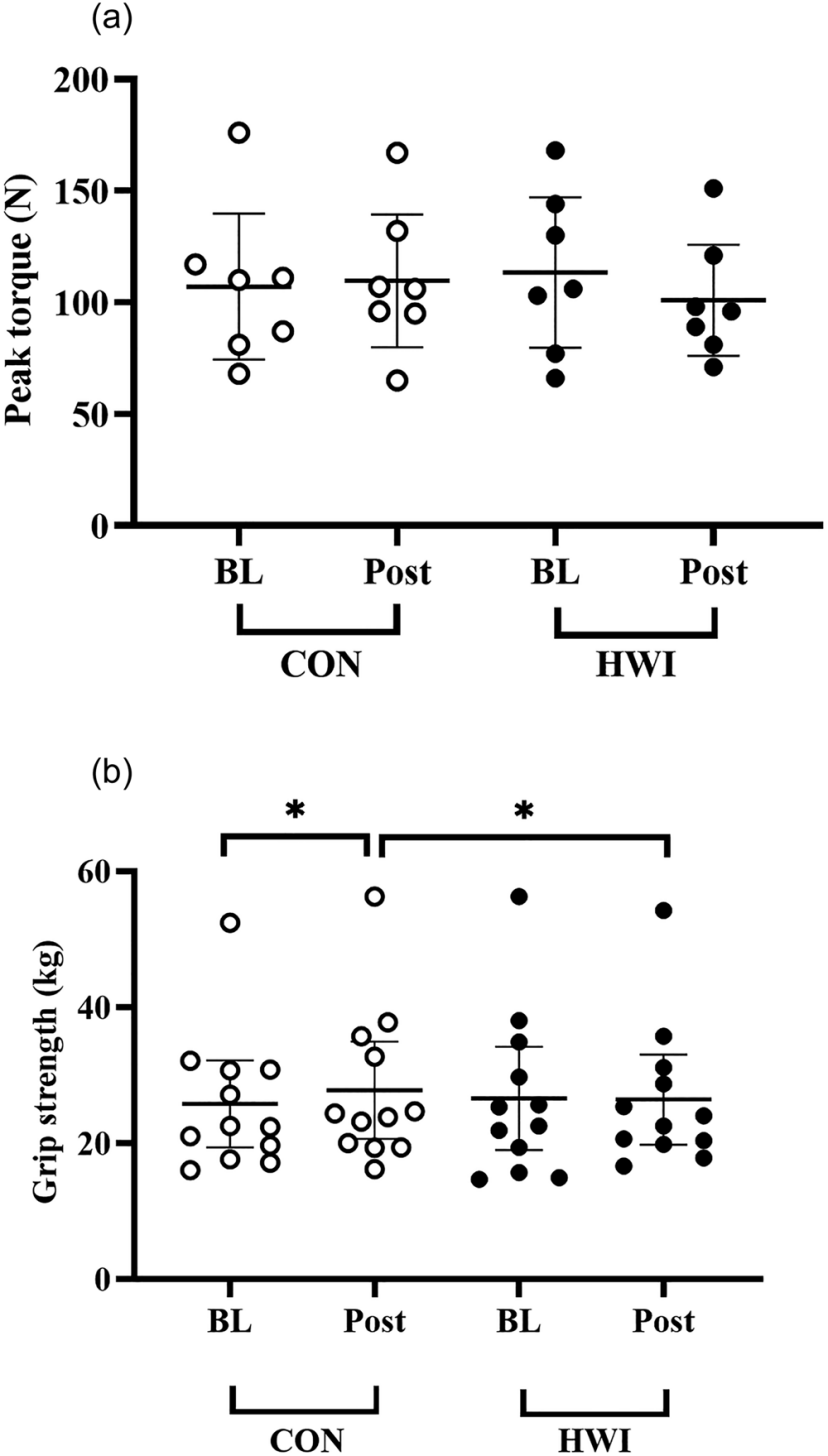
Peak quadriceps and grip strength at baseline (BL) and post for control (CON) and hot water immersion arms (HWI). Individual data points with means and 95% confidence intervals for peak quadriceps torque (*n* = 7) (a) and grip strength (*n* = 12) (b) at baseline (BL) and post for control (CON) and hot water immersion arm (HWI). Open circles represent the CON arm and filled circles represent the HWI arm. Data were analysed using a two‐way repeated measures linear mixed model. *P < 0.05 significant difference.

**FIGURE 4:**
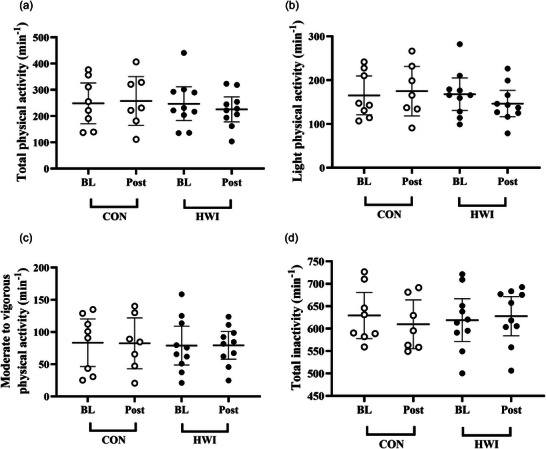
Physical activity accelerometry data. Individual data points with means and 95% confidence intervals for accelerometry data for: total physical activity (a), light physical activity (b), moderate to vigorous inactivity (c), and total inactivity (d) (*n* = 10) at baseline (BL) and post for control (CON) and hot water immersion arms (HWI). Open circles represent pre‐HWI and filled circles represent the HWI arm. Data were analysed using a two‐way linear mixed model.

There was no change in the primary outcome, peak quadriceps torque following repeated HWI (interaction: mean difference: −15.00 N m; 95% CI: −34.67 to 4.67; *F*_(1,18)_ = 2.566, *P* = 0.127, η^2^_p_ = 0.125). There was a significant interaction for grip strength (mean difference: −2.18 kg; 95% CI: −4.05 to 0.32; *F*_(1,44)_ = 5.585, *P* = 0.023, η^2^_p_ = 0.113), with *post hoc* tests revealing an increase at CON post compared to CON baseline (*P* = 0.004), while HWI post was lower than CON post (*P* = 0.039) (Figures 3 and 4).

### 3.2 Short physical performance battery

Repeated HWI had no effect total SPPB (interaction: mean difference: −0.6; 95% CI: −0.2 to 1.4; *F*_(1,44)_ = 2.039, *P* = 0.160, η^2^_p_ = 0.044), balance (mean difference: 0.3; 95% CI: −0.1 to 0.7; *F*_(1,44)_ = 2.694, *P* = 0.108, η^2^_p_ = 0.058), 4‐m walk (mean difference: 0.0; 95% CI: −0.2 to 0.2; *F*_(1,44)_ = 0.000, *P* = 1.000, η^2^_p_ = 0.000) or STS scores (mean difference: 0.3; 95% CI: −0.5 to 1.0; *F*_(1,44)_ = 0.488, *P* = 0.489, η^2^_p_ = 0.011). Repeated HWI had no effect on times for tandem balance (interaction: mean difference: 1.64 s; 95% CI: −0.28 to 3.56; *F*_(1,44)_ = 2.958, *P* = 0.092, η^2^_p_ = 0.063), 4‐m walk (mean difference: −0.09 s; 95% CI: −0.54 to 0.35; *F*_(1,44)_ = 0.175, *P* = 0.678, η^2^_p_ = 0.004) or STS times (mean difference: −0.04 s; 95% CI: −1.88 to 1.80; *F*_(1,44)_ = 0.002, *P* = 0.968, η^2^_p_ = 0.000) (Tables 2 and 3).

**TABLE 2:** Short physical performance battery results (*n* = 12).

Parameter	CON BL	CON post	HWI BL	HWI post
Total score	10.8 ± 1.2	10.8 ± 1.5	10.8 ± 1.2	11.4 ± 0.9
Balance (score)	3.8 ± 0.6	3.6 ± 0.8	3.7 ± 0.7	3.8 ± 0.6
4‐m walk (score)	3.8 ± 0.4	3.8 ± 0.6	3.9 ± 0.3	3.9 ± 0.3
Sit to stand (score)	3.2 ± 0.8	3.4 ± 0.7	3.2 ± 1.0	3.7 ± 0.5
Tandem balance time (s)	8.87 ± 2.98	7.94 ± 3.95	8.45 ± 3.24	9.17 ± 2.89
4‐m walk time (s)	3.71 ± 0.84	3.78 ± 1.05	3.68 ± 0.70	3.66 ± 0.73
Sit to stand time (s)	12.14 ± 2.97	11.14 ± 1.69	11.90 ± 2.58	10.85 ± 1.67

*Note*: Total short physical performance battery scores out of 12; individual component (balance, 4‐m walk, sit to stand) scores out of 4. Abbreviations: BL, baseline; CON, control; HWI, hot water immersion.

**TABLE 3:** Knee extension kinetics and gait measures (*n* = 11).

Parameter	CON BL	CON Post	HWI BL	HWI post
Knee extension kinetics				
Knee extension velocity (° s^−1^)	195.24 ± 57.53	202.98 ± 61.62	194.45 ± 55.61	204.28 ± 44.33
Knee extension moment (N m kg^−1^)	0.85 ± 0.26	0.78 ± 0.18	0.76 ± 0.20	0.78 ± 0.21
Knee extension power (W kg^−1^)	1.83 ± 1.18	1.61 ± 0.94	1.37 ± 0.63	1.53 ± 0.86^*^
Gait measures				
Stride length (m)	1.35 ± 0.17	1.35 ± 0.20	1.36 ± 0.20	1.32 ± 0.20
Stride frequency (strides s^−1^)	0.88 ± 0.08	0.87 ± 0.07	0.88 ± 0.06	0.88 ± 0.06
Stride velocity (m s^−1^)	1.20 ± 0.20	1.18 ± 0.19	1.19 ± 0.20	1.17 ± 0.22

* *P *< 0.05 significant main effect of condition for knee extension power. Abbreviations: BL, baseline; CON, control; HWI, hot water immersion.

### 3.3 Motion capture

Repeated HWI had no effect on knee extension velocity (interaction: mean difference: 2.08° s^−1^; 95% CI: 29.00–33.16; *F*_(1,30)_ = 0.019, *P* = 0.892, η^2^_p_ = 0.001), knee extension moment (mean difference: 0.07 N m kg^−1^; 95% CI: −0.08 to 0.22; *F*_(1,25.32)_ = 1.039, *P* = 0.318, η^2^_p_ = 0.039) or knee extension power (mean difference: 0.37 W kg^−1^; 95% CI: −0.10–0.83; *F*_(1,35)_ = 2.569, *P* = 0.118, η^2^_p_ = 0.068). Repeated HWI had no effect on stride length (interaction: mean difference: −0.01 m; 95% CI: −0.06 to 0.04; *F*_(1,39)_ = 0.183, *P* = 0.671, η^2^_p_ = 0.004), stride frequency (mean difference: 0.01 strides s^−1^; 95% CI: −0.02 to 0.04; *F*_(1,29.017)_ = 0.662, *P* = 0.422, η^2^_p_ = 0.022) or stride velocity (mean difference: 0.01 m s^−1^; 95% CI: −0.07–0.08; *F*_(1,39)_ = 0.040, *P* = 0.843, η^2^_p_ = 0.001).

### 3.4 Physical activity levels

Repeated HWI had no effect on total PA (interaction: mean difference: −26.92 min; 95% CI: –75.50 to 21.66; *F*_(1,22.244)_ = 1.319, *P* = 0.263, η^2^_p_ = 0.056), light intensity PA (mean difference: –26.73 min; 95% CI: –56.61 to 3.15; *F*_(1,22.222)_ = 3.437, *P* = 0.077, η^2^_p_ = 0.133), moderate to vigorous PA (mean difference: −0.16 min; 95% CI: –26.21 to 25.89; *F*_(1,22.340)_ = 0.000, *P* = 0.990, η^2^_p_ = 0.000) or total inactivity (mean difference: –24.79 min; 95% CI: –42.89 to 92.47; *F*_(1,22.723)_ = 0.575, *P* = 0.456, η^2^_p_ = 0.025).

## 4 DISCUSSION

This is the first study to assess the effects of 6 weeks of repeated HWI on strength, lower extremity power and function, and physical activity in healthy older adults. The principal novel findings of this study are that 6 weeks of repeated HWI did not improve quadriceps strength, grip strength, performance in the SPPB, knee power, gait characteristics or weekly physical activity quantities. The results indicate that HWI does not affect strength and physical function in healthy older adults. Possible explanations for the lack of effect could include the stimulus intensity, the length of intervention and baseline physical function in our study population.

### 4.1 Strength

Six weeks of HWI had no effect on peak quadriceps torque in our cohort of healthy older adults. These findings are in contrast to previous repeated PHT studies showing improvements in quadriceps strength in young, healthy (Kim et al., 2020) and middle‐aged adults (Goto et al., 2011), but agree with recent findings in older adults (Fuchs et al., 2024; Ro et al., 2025). Purported mechanisms for the lack of improvement in quadriceps strength could be muscle protein synthesis or muscle size, which previously did not change in healthy older adults following 8 weeks of PHT (Fuchs et al., 2024). A lack of improvement in leg strength could also be related to changes in muscle temperature. Previous studies showing increased leg strength applied local heating stimuli at a higher temperature (>50°C) or intensity, increasing muscle temperature by 3.3°C (Goto et al., 2011), 4.2°C (Hafen et al., 2019), 4.8°C (Denny et al., 2025) and >42°C leg‐HWI (2.4–2.8°C (Rodrigues et al., 2020b, 2023). Therefore, localised heating at a higher temperature may have the capacity to provide a greater effect when targeting specific muscle groups (e.g. quadriceps) than what is safely feasible with whole‐body HWI protocols in older humans.

This is the first study to investigate the effects of repeated HWI on grip strength in older adults. We showed that 6 weeks of repeated HWI had no effect on grip strength in healthy older adults. Our findings are in contrast to previous repeated PHT (Fuchs et al., 2024) and exercise (Labott et al., 2019) studies showing increased grip strength in older adults. Fuchs et al. (2024) similarly observed no concurrent changes in leg strength, and therefore any mechanistic difference between their findings and the present study is unclear and requires further investigation, though a learning effect could have occurred in their pre–post study design. Labott et al. (2019) proposed that targeted upper body exercises (i.e. resistance training) within exercise protocols likely elicited improvements, suggesting that HWI, which provided a passive stimulus, may not have been sufficient to increase grip strength. It should be noted that whilst not a primary outcome measure, the study by Fuchs et al. (2024) and the present study likely suffered from a lack in statistical power for grip strength. Indeed, data from Fuchs et al. (2024) indicates that a definitive trial with grip strength as the primary outcome, an effect size of 0.197, and 80% power would require 205 participants.

### 4.2 Power, lower extremity function and gait

We showed that repeated HWI had no effect on knee extension power during the STS exercise. Our results are in contrast to previous mechanistic findings testing acute (Mornas et al., 2021) and repeated (Racinais et al., 2017) whole‐body PHT in young, healthy individuals which demonstrated improved contractile function in the leg muscle. Repeated HWI also did not significantly alter the total score or any individual components of the SPPB. These findings are in contrast to previous work in older adults showing a significant increase in total SPPB score following a 6 month exercise intervention (Gudlaugsson et al., 2012), but similar to Ro et al. (2025), who saw no change in STS times following 12 weeks of PHT in healthy older adults. It is therefore plausible that the length of the intervention or stimulus (i.e. compared to exercise) in the present study was not sufficient to yield improvements to knee power or the SPPB. It should also be noted that baseline total SPPB scores in the present study were in the high range (10–12), indicating little to no mobility impairment, and our participants’ capacity to improve their scores may have been low. A lack of improvement in lower extremity function could also have been influenced by a ceiling effect within the SPPB, which may mask deficits or improvements in high‐functioning older adults (i.e. our participants) (Boulgarides et al., 2003). Thus, the effects of repeated HWI could be more applicable to lower‐functioning (i.e. with impaired mobility) older adults and merit further investigation.

Gait characteristics during walking were not different in any of the visits. This aligns with our findings that repeated HWI did not alter 4‐m walk score or time in the SPPB, and the lack of improvement in quadriceps strength. Whilst chronic exercise can improve gait characteristics (Hortobágyi et al., 2015), it has been suggested that increases in muscle strength through progressive resistance training, and not aerobic exercise, are the most necessary to improve gait speed in older adults (Van Abbema et al., 2015). Given we and others showed no increases in strength, PHT may not be an effective intervention to improve gait for healthy older adults.

### 4.3 Physical activity

Repeated HWI had no effect on total PA or different intensities of PA across the final 7‐day period of the HWI arm compared to baseline or the CON arm. This is in contrast to previous work showing increases in PA in older adults following chronic exercise (Gudlaugsson et al., 2012). As HWI mimics some of the cardiometabolic effects of light intensity exercise (James et al., 2021), our intervention could have acted as a replacement for activity that participants performed in other 7‐day blocks, rather than as an additional source of PA. In contrast, however, Fuchs et al. (2024) observed a decrease in light intensity PA in older adults after 8 weeks of sauna usage, despite no changes in total, moderate or vigorous PA. It should be noted that PA data in the study of Fuchs et al. was obtained through self‐reported questionnaires, which can both under‐ and over‐report PA levels compared to device‐based accelerometry data (Prince et al., 2008). Therefore, HWI may not serve as a suitable method to increase overall PA, but could be an appropriate alternative to limit declines in overall activity for individuals who are unwilling or unable to exercise.

### 4.4 Strengths and limitations

The study design (a randomised, crossover, control trial) is a key strength of the present study, which is the first to assess muscle function in older adults following repeated HWI. The study population is an additional strength, as previous work assessing muscle function following PHT has primarily focused on younger populations with no apparent functional declines from ageing. Some limitations, however, warrant discussion. As the present study was part of a wider trial, our results likely lack adequate statistical power and should be interpreted with caution. Additionally, only seven participants out of 12 completed testing on the IKD due to equipment failure during the experimental trial, and some motion capture for knee extension kinetics trials was missing, causing unbalanced data between study arms, which could have skewed our data for knee power.

Due to laboratory space constraints and mobility issues entering and exiting the hot tub, two different vessels were used for HWIs. This could have impacted body temperatures during HWI sessions, as we were unable to manipulate body positions in the hot tub as effectively as the immersion tank, where participants in the hot tub were raised out in stepwise fashion rather than gradually on a pulley. A further limitation is that the CON arm consisted of no intervention, whereas a thermoneutral sham could have helped to account for the effects of hydrostatic squeeze. Participants were also instructed to maintain their current routines during the CON arm and 6‐week washout, but we are unable to preclude that participants undertook new forms of activity or exercise during these periods, despite instructions to avoid this. The absence of a change in PA levels at all intensities at the beginning and end of the CON arm, however, would suggest that participants observed these instructions, and the significant findings in grip strength were the result of a type I error. However, no significant order effects were detected for any outcome measure.

### 4.5 Conclusion

The present study is the first to assess the effects of repeated HWI on strength, power, lower extremity function and physical activity levels in healthy older adults. Overall, repeated HWI did not alter quadriceps strength, grip strength, knee power, lower extremity function or PA levels. Our findings suggest that repeated HWI may not be an effective method to improve indices of muscle function in healthy older adults. Future work is warranted testing longer interventions and in clinical populations who are frail or have impaired mobility and may be limited in their ability to perform exercise.

## AUTHOR CONTRIBUTIONS

Daniel D. Piccolo, Anthony I. Shepherd, Jo Corbett and Zoe L. Saynor conceived and designed the research. Daniel D. Piccolo, Anthony I. Shepherd, Mohammad G. A. Alnajjar, Luke Hudson, Poppy A. Marsh and Veronika Praskacova collected the data. Daniel D. Piccolo, Anthony I. Shepherd, Timothy A. Exell, Joseph M. Moore, Amy Wright, Melitta A. McNarry, and Kelly A. Mackintosh analysed the data. Daniel D. Piccolo, Anthony I. Shepherd, Jo Corbett and Zoe L. Saynor interpreted results of experiments and trial. Daniel D. Piccolo and Anthony I. Shepherd prepared figures. Daniel D. Piccolo, Anthony I. Shepherd, Jo Corbett and Zoe L. Saynor drafted the manuscript. Daniel D. Piccolo, Anthony I. Shepherd, Jo Corbett and Zoe L. Saynor, Timothy A. Exell, Joseph M. Moore, Amy Wright, Mohammad G. A. Alnajjar, Luke Hudson, Poppy A. Marsh, Veronika Praskacova, Melitta A. McNarry, and Kelly A. Mackintosh edited and revised the manuscript. All authors approved the final version of manuscript and agree to be accountable for all aspects of the work in ensuring that questions related to the accuracy or integrity of any part of the work are appropriately investigated and resolved. All persons designated as authors qualify for authorship, and all those who qualify for authorship are listed.

## CONFLICT OF INTEREST

None declared.

## Data Availability

The full anonymised dataset has been made freely available as supplementary material on our university repository (https://doi.org/10.17029/40adb0f3‐23ed‐48c0‐9cd3‐ff7083c9d2f4). For the purpose of open access, the author(s) has applied a Creative Commons Attribution (CC‐BY) licence to any author accepted manuscript version arising from this submission.

## Appendix

**TABLE A1:** Main effects for parameters of strength.

Parameter	Main effect of condition					Main effect of time				
	*F*	*P*	Mean difference	CI	η^2^_p_	*F*	*P*	Mean difference	CI	η^2^ *_p_*
Peak torque (N m)	0.067	0.798	−1.21	−11.05 to 8.62	0.004	1.108	0.306	−4.93	−14.77 to 4.91	0.058
Grip strength (kg)	0.422	0.519	−0.30	−1.29 to 0.69	0.01	3.938	0.053	0.92	−0.74 to 1.91	0.082

Main effects for condition and time with 95% confidence intervals (CI) plus order effects for peak torque (*n *= 7) and grip strength (*n* = 12). Data were analysed using a two‐way repeated measures linear mixed model for parametric data or generalised linear mixed model for non‐parametric data.

**TABLE A2:** Main effects for the short physical performance battery.

Parameter	Main effect of condition					Main effect of time				
	*F*	*P*	Mean difference	CI	η^2^_p_	*F*	*P*	Mean difference	CI	η^2^_p_
Total score	2.039	0.160	0.3	−0.1 to 0.7	0.044	3.371	0.073	0.4	0.0 to 0.8	0.071
Balance (score)	0.673	0.416	0.1	−0.1 to 0.3	0.015	0.000	1.000	0.0	−0.2 to 0.2	0.000
4‐m walk (score)	1.941	0.171	0.1	0.0 to 0.2	0.042	0.000	1.000	0.0	−0.1 to 0.1	0.000
Sit to stand (score)	0.488	0.489	0.1	−0.2 to 0.5	0.011	4.389	0.042*	0.4	0.0 to 0.7	0.091
Tandem balance time (s)	0.728	0.398	0.407	−0.58 to 1.39	0.016	0.049	0.827	−0.105	−1.09 to 0.88	0.001
4‐m walk time (s)	0.507	0.480	−0.079	−0.30 to 0.14	0.011	0.043	0.837	0.023	−0.20 to 0.24	0.001
Sit to stand time (s)	0.339	0.563	−0.266	−1.17 to 0.64	0.007	4.931	0.032*	−1.014	−1.92 to −0.11	0.101

Main effects for condition and time with 95% confidence intervals (CI) plus order effects for the Short Physical Performance Battery. Data were analysed using a two‐way repeated generalised linear mixed model.

* *P *< 0.05 significant main effect of time.

**TABLE A3:** Main effects for knee extension kinetics and gait characteristics.

Parameter	Main effect of condition					Main effect of time				
	*F*	*P*	Mean difference	CI	η^2^_p_	*F*	*P*	Mean difference	CI	η^2^_p_
Knee extension kinetics										
Knee extension velocity (deg s^−1^)	0.001	0.974	0.253	−15.287 to 15.793	0.000	1.332	0.257	8.783	−6.756 to 24.323	0.043
Knee extension moment (N m kg^−1^)	0.782	0.385	−0.032	−0.108 to 0.043	0.030	0.293	0.593	−0.019	−0.093 to 0.055	0.011
Knee extension power (W kg^−1^)	4.041	0.052	−0.241	−0.486 to 0.003	0.104	0.317	0.577	−0.049	−0.287 to 0.190	0.009
Gait measures										
Stride length (m)	0.001	0.979	0.001	−0.026 to 0.027	0.000	0.167	0.685	−0.005	−0.031 to 0.021	0.004
Stride frequency (strides s^−1^)	0.006	0.940	−0.001	−0.015 to 0.014	0.000	1.306	0.263	−0.008	−0.022 to 0.006	0.043
Stride velocity (m s^−1^)	0.003	0.957	−0.001	−0.037 to 0.035	0.000	0.771	0.385	−0.016	−0.052 to 0.020	0.019

Main effects for condition and time with mean differences and 95% confidence intervals (CI) for knee extension kinetics and gait measures. Data were analysed using a two‐way repeated measures linear mixed model for parametric data and generalised linear mixed model for non‐parametric data.

**TABLE A4:** Main effects for accelerometry data.

Parameter	Main effect of condition					Main effect of time				
	*F*	*P*	Mean difference	CI	η^2^_p_	*F*	*P*	Mean difference	CI	η^2^_p_
Total physical activity (min^−1^)	0.492	0.490	−8.334	−32.958 to 16.289	0.022	0.440	0.514	−7.772	−32.063 to 16.518	0.019
Light physical activity (min^−1^)	0.459	0.505	−4.954	−20.099 to 10.191	0.020	1.270	0.272	−8.124	−23.066 to 6.818	0.054
Moderate to vigorous physical activity (min^−1^)	0.289	0.596	−3.425	−16.618 to 9.769	0.013	0.003	0.957	0.339	−12.685 to 13.363	0.000
Total inactivity (min^−1^)	0.277	0.604	8.686	−25.425 to 42.796	0.012	0.045	0.833	−3.483	−37.321 to 30.356	0.002

Main effects for condition and time with 95% confidence intervals (CI) plus order effects for accelerometry data. Data were analysed using a two‐way repeated measures linear mixed model.

**TABLE A5:** Order effects for parameters of strength.

Parameter	*F*	*P*	η^2^_p_
Peak torque	0.639	0.599	0.096
Grip strength	1.261	0.300	0.079

Data were analysed using a one‐way repeated measures linear mixed model for parametric data and generalised linear mixed model for non‐parametric data.

**TABLE A6:** Order effects for the short physical performance battery.

Parameter	*F*	*P*	η^2^_p_
Total score	1.247	0.304	0.078
Balance	1.122	0.350	0.071
4‐m walk	0.647	0.589	0.042
Sit to stand	1.461	0.238	0.091
Tandem balance time	1.107	0.356	0.070
4‐m walk time	0.172	0.915	0.012
Sit to stand time	2.037	0.122	0.122

Data were analysed using a one‐way repeated measures linear mixed model for parametric data or generalised linear mixed model for non‐parametric data.

**TABLE A7:** Order effects for knee extension kinetics and gait measures.

Parameter	*F*	*P*	η^2^_p_
Knee extension kinetics			
Knee extension velocity	0.625	0.604	0.059
Knee extension moment	0.236	0.871	0.027
Knee extension power	1.787	0.168	0.133
Gait measures			
Stride length	1.07	0.373	0.076
Stride frequency	0.497	0.687	0.049
Stride velocity	0.602	0.618	0.044

Data were analysed using a one‐way repeated measures linear mixed model for parametric data and generalised linear mixed model for non‐parametric data.

**TABLE A8:** Order effects for accelerometry data.

Parameter	*F*	*P*	η^2^_p_
Total physical activity	0.279	0.840	0.036
Light physical activity	0.644	0.595	0.080
Moderate to vigorous physical activity	0.083	0.969	0.011
Total inactivity	0.878	0.467	0.104

Data were analysed using a one‐way repeated measures linear mixed model for parametric data or generalised linear mixed model for non‐parametric data.
